# Mechanisms and Approaches for Overcoming Enzalutamide Resistance in Prostate Cancer

**DOI:** 10.3389/fonc.2018.00180

**Published:** 2018-05-24

**Authors:** Alexandra Vander Ark, Jingchen Cao, Xiaohong Li

**Affiliations:** Program for Skeletal Disease and Tumor Microenvironment, Center for Cancer and Cell Biology, Van Andel Research Institute, Grand Rapids, MI, United States

**Keywords:** enzalutamide resistance, castration-resistant prostate cancer, androgen receptor mutants, androgen receptor splice variants, bypass, intratumoral androgen biosynthesis, lineage plasticity, tumor microenvironment

## Abstract

Enzalutamide, a second-generation small-molecule inhibitor of the androgen receptor (AR), has been approved for patients who failed with androgen deprivation therapy and have developed castration-resistant prostate cancer. More than 80% of these patients develop bone metastases. The binding of enzalutamide to the AR prevents the nuclear translocation of the receptor, thus inactivating androgen signaling. However, prostate cancer cells eventually develop resistance to enzalutamide treatment. Studies have found resistance both in patients and in laboratory models. The mechanisms of and approaches to overcoming such resistance are significant issues that need to be addressed. In this review, we focus on the major mechanisms of acquired enzalutamide resistance, including genetic mutations and splice variants of the AR, signaling pathways that bypass androgen signaling, intratumoral androgen biosynthesis by prostate tumor cells, lineage plasticity, and contributions from the tumor microenvironment. Approaches for overcoming these mechanisms to enzalutamide resistance along with the associated problems and solutions are discussed. Emerging questions, concerns, and new opportunities in studying enzalutamide resistance will be addressed as well.

## Introduction

Of the estimated 26,000 prostate cancer (PCa) deaths in 2016 in the United States, over 80% involved bone metastases (1–3). Second-line hormonal therapies such as enzalutamide (also known as MDV-3100) improve overall patient survival only by several months in about 50% of the patients, and almost all patients develop drug resistance (4–8). There is an urgent need to determine the mechanisms of drug resistance, identify new approaches for overcoming such resistance, and from that knowledge, develop better treatments for PCa bone metastasis.

**Figure 1:**
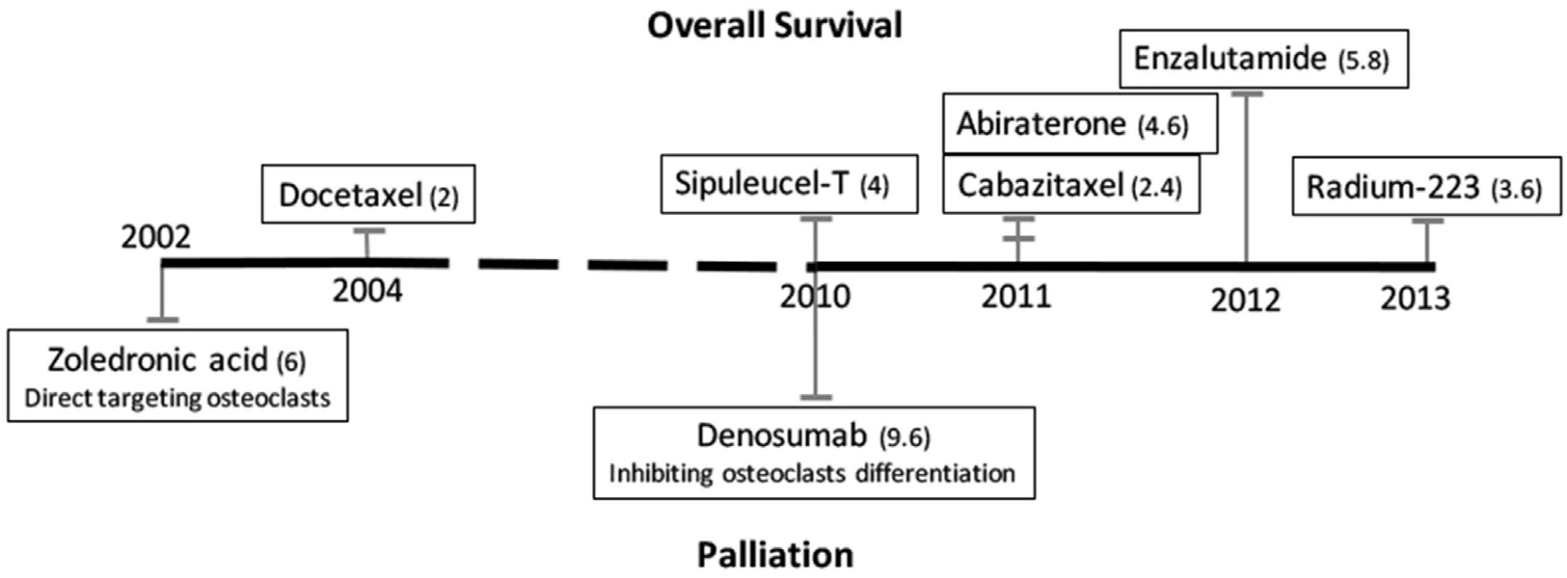
Time line of FDA-approved treatments for patients with castration-resistant prostate cancer bone metastases. Treatments that improve patients’ overall survival are listed above the black line, and palliation treatments are listed below. The numbers in parentheses are median months of overall increased survival or median months delayed to first detection of skeletal-related events, respectively.

The first-line treatment option for men with PCa is hormonal therapy. Huggins and Hodges discovered in 1941 that removing androgens or providing estrogens could inhibit the progression of PCa (9). Despite the initial response of most patients to androgen deprivation therapy (ADT), the disease typically progresses to a castration-resistant state within 18–24 months (10, 11). Castration-resistant prostate cancer (CRPC) is defined by disease progression despite hormonal therapies, which is often indicated by an increase of prostate-specific antigen (PSA), a target of androgen signaling activation. As the disease progresses, the CRPC ultimately metastasizes to bone and later to other organs. Patients with metastatic CRPC (mCRPC) have a poor prognosis and a predicted survival of fewer than 2 years from the initial time of progression; such patients account for a large portion of the PCa deaths per year (2, 3, 12, 13). In 2012, enzalutamide, which significantly improved patient survival, was approved. The time line of FDA-approved treatments and their respective effects are shown in Figure 1 (5, 6, 14, 15). However, the survival benefits of enzalutamide were achieved in only up to 50% of PCa patients treated, and patients who initially responded eventually developed resistance (4). Studies of the mechanisms of intrinsic and acquired resistance led to approaches to overcome it.

## Enzalutamide Function at the Molecular Level

The androgen receptor (AR) is a transcription factor and a member of the nuclear receptor superfamily. It consists of an N-terminal transactivation domain (exon 1), a central DNA-binding domain (DBD; exons 2–3), a C-terminal ligand-binding domain (LBD; exons 4–8), and a hinge region between the DBD and LBD that is involved in nuclear localization and degradation (16). The N terminus has a unique LxxLL-like motif, which binds to a hydrophobic cleft of the C terminus that is generated by ligand binding to the receptor. This binding also stabilizes the ligand binding-caused N terminal–C terminal physical interaction of the receptor. The N–C interaction is initially intramolecular in the cytoplasm and is necessary for nuclear localization, but the interaction changes to intermolecular in the nucleus (17–22).

Androgen receptor antagonists all bind to the LBD (23). Binding of the first-generation antagonists (such as flutamide or bicalutamide) does not prevent the intra- or intermolecular N–C interactions (19). Thus, after binding of these antagonists, the AR still translocates to the nucleus, binds to chromatin, and acts as an agonist. This antagonist to agonist switch is partially due to the highly conserved overall configurations of LBD binding with ligand (testosterone or dihydrotestosterone), bicalutamide, or flutamide (24). Enzalutamide binds to the AR with an eightfold higher affinity than bicalutamide (25). Furthermore, no intramolecular N–C interaction was found upon binding of enzalutamide, which blocks the translocation of the receptor to the nucleus and therefore blocks subsequent signaling activation (20).

## Enzalutamide Resistance in PCa Cells and Approaches to Overcoming IT

Because enzalutamide binds to the AR LBD, the presence of splicing variants that lack the LBD and of constitutively activated AR in PCa cells apparently confer the intrinsic resistance to enzalutamide. AR-V7 in circulating tumor cells (CTC) has been associated with enzalutamide resistance in PCa patients, although larger-scale studies are needed to conclude that AR-v7 is the cause of resistance (26–30). On the other hand, the overwhelming majority of enzalutamide-treated patients who have robust declines in PSA eventually develop resistance, with increasing PSA and/or progression of bone lesions that suggest acquired resistances. We discuss several major mechanisms of acquired resistance in the following sections, based on the original research publication and recent reviews (21, 31–33).

### AR Mutations and Splice Variants (AR-V)

One of the AR mutant F876L (substitution of phenylalanine for leucine at the 876 position), which confers enzalutamide resistance *in vitro* and *in vivo* (34, 35), spontaneously emerged among enzalutamide-resistant clones. These clones appeared after weeks of continuous enzalutamide treatments of PCa cell lines with AR either in culture or xenografted in immune comprised mice. The F876L mutation is in the LBD and is adjacent to the homozygous T877A mutation in LNCaP cells. The F876L mutation is heterozygous, with a mutant allelic mRNA frequency of approximately 40% in patient samples. Mechanistically, a positive correlation of increased AR activation and E2F1 activation was found in these resistant clones, correspondently, cyclin-dependent kinase (CDK) 4/6 inhibitor has been tested as an approach to overcoming F876L-based resistance. However, the specificity of the inhibitor is a concern, because of the key role of CDK4/6 in cell cycle and because one of the CDK4/6 inhibitors, LEE011, was shown to significantly reduce the viability of both parental and enzalutamide-resistant cells (34).

Other studies have focused on developing novel small-molecule inhibitors targeting the AR mutants. One example is darolutamide (ODM-201) (36), an AR antagonist. Darolutamide significantly inhibited the growth of an enzalutamide-resistant PCa clone such as the LNCaP-derived MR49F cell line that has the F877L mutation *in vitro* and *in vivo*. Mechanistically, darolutamide was able to inhibit the transcriptional activity of three AR mutants that confer enzalutamide resistance, such as F877L, F877L/T878A, and H875Y/T878A. Darolutamide has a different chemical structure than enzalutamide, and *in silico* simulation of the drug’s mode of action showed a distinct structure of darolutamide binding with the AR.

Another development is an AR degradation enhancer, ASC-J9 [5-hydroxy-1,7-bis(3,4-dimethoxyphenyl)-1,4,6-heptatrien-3-one] (37–39). ASC-J9 suppressed enzalutamide-resistant PCa progression (40), and it degrades not only wild-type AR but also AR-F876L and splice variants of such as AR-v7 (41–45). ASC-J9 degrades the AR by enhancing its association with the murine double minute protein 2 (MDM2) (41), an E3 ubiquitin ligase that drives AR clearance *via* proteasome-mediated degradation. However, this has not been confirmed by rescuing the AR degradation using proteasome inhibitors. It is not known whether the same mechanism applies for the downregulation of AR-F877L by ASC-J9. This is possible, because one amino acid mutation may completely change the AR structure and endow it with enzalutamide resistance but still allow it to interact with MDM2. ASC-J9 was also shown to have additional effects that contribute to the inhibitions of PCa progression and metastasis. For example, in PCa cells, ASC-J9 was able to suppress expression fatty acid synthase that stimulates PCa cell growth and invasion (46) or to inhibit the phosphorylation of STAT3 that promotes PCa stem/progenitor cells invasion (39); in the cells of the tumor microenvironment, ACS-J9 could suppress the CD4+ T cell migration that contribute to the progression of prostatitis (47), or the macrophage infiltration that stimulated PCa cell invasion (38). Together, these studies suggested that ACS-J9 was very promising in battling PCa. However, its broader effects on other signaling pathways and on cells from the microenvironment need to be explored further before being translated to clinical trials.

Furthermore, BET [bromodomain (BRD) and extraterminal] inhibitors, such as JQ1 and OTX015, have been shown to overcome enzalutamide resistance of CRPC conferred by AR-V (48, 49). BET inhibitors target the amino-terminus of BRD proteins, BRD4, and exhibit proliferation inhibition in a wide range of cancers, including CRPC. The underlying mechanistic studies suggested that JQ1 inhibited expression of full-length AR and AR-V, thus overcoming enzalutamide resistance (50). However, BET inhibitors as single agents may not succeed in the clinic because of a recent study showing acquired resistance to BET inhibitors in CRPC cells (51). It is not known whether the combination of BET inhibitors with enzalutamide induce resistance, or whether combination of BET inhibitors with CDK9 and/or PARP (poly ADP ribose polymerase) inhibitors overcomes enzalutamide resistance.

In summary, the above approaches target the outcomes of enzalutamide resistance. We believe targeting the causes of drug resistance will be most effective, which will require answers to pivotal questions such as: How does enzalutamide treatment induce mutations of AR? and What types of PCa cells will develop enzalutamide resistance?

### Bypassing Signaling Pathways

The bypassing of AR signaling through increasing glucocorticoid receptor (GR) expression at both the mRNA and protein levels has been identified in enzalutamide-resistant PCa cells, and increases of GR have been confirmed in clinical samples (52, 53). The activation of the GR by dexamethasone is sufficient to confer enzalutamide resistance, and a novel GR antagonist, arylpyrazole compound 15, can restore sensitivity to the drug (52–54). However, blocking glucocorticoid signaling is neither practical nor effective, because glucocorticoid is essential for life, and also because GR antagonists activate AR target genes (52). Therefore, researchers have focused on how enzalutamide increases glucocorticoid signaling. One recent study (55) found that enzalutamide treatment sustained the level of cortisol, the active ligand for the GR, thus elevating glucocorticoid signaling and producing a loss of 11β-hydroxysteroid dehydrogenase-2 (11β-HSD2), the enzyme that converts cortisol to the inactive form, cortisone. It was further shown that 11β-HSD2 loss was mediated by an increase of AMFR, an ubiquitin E3-ligase autocrine mobility factor receptor. Approaches such as overexpression of 11β-HSD2 or knock-down of AMFR were effective in reversing enzalutamide resistance. Future research will need to address how to translate these approaches to therapies for patients.

Androgen signaling not only activates but also suppresses various downstream target genes, some of which are oncogenic, such as c-Met (56), c-Myb (57), or enhancer of zeste homolog 2 (EZH2) (58). Blocking either any of these proteins or their respective downstream targets was shown to inhibit castration-resistant PCa cell proliferation (*in vitro*) or growth (*in vivo*). Logically, since enzalutamide treatment induces c-Met, c-Myb, and EZH2 or activates the target genes, the combination of enzalutamide with inhibition of each of these targets may have better efficacy. However, further preclinical and clinical testing are needed for confirmation and validation.

On the other hand, enzalutamide treatment was shown to activate PI3K/AKT signaling by reducing FK506 binding protein 5 (a chaperone for the PHLPP) that destabilizes PHLPP (PH domain and leucine rich repeat protein phosphatases), the AKT phosphatase (59). Combining enzalutamide with BEZ235 (a dual PI3K and mTORC1/2 inhibitor) or AKT1/2 inhibitor led to significant inhibition of PCa tumor growth in mice (59). Another study showed increased noncanonical Wnt signaling, independent of increased GR, in CTC obtained from enzalutamide-resistant PCa patients compared with enzalutamide-naïve PCa patients by analyzing the RNA sequences of the single CTC (60). Furthermore, increased Wnt5A was confirmed to represent the noncanonical Wnt signaling in CTC, as well as in enzalutamide-treated or enzalutamide-resistant LNCaP PCa cells (60). However, the effectiveness of inhibiting PCa growth by combining enzalutamide with medicinal approaches for blocking noncanonical Wnt signaling or Wnt5A has not been tested. This avenue to overcoming enzalutamide resistance is practical since the Wnt5a antagonist has been tested in clinical trials in breast and colorectal cancers (61). Enzalutamide treatment has also been shown to increase autophagy, in which the AMPK pathway was upregulated (62). Combinations of enzalutamide with autophagy inhibitors enhanced the therapeutic response, particularly when using clomipramine or metformin, both of which are FDA approved as antidepressant and antidiabetic drugs, respectively. However, increasing autophagy is a general cause by blocking AR signaling, because the same effect was also observed in bicalutamide-resistant cells (62).

Other bypass pathways have also been reported. The associations of β-catenin, miRNA16, and oncostatin M with enzalutamide resistance were identified by gene profiling of parental cells versus enzalutamide-resistant cells (31). Correlations between each of these and CRPC cells had been reported in various studies (63–67). However, the effectiveness of overcoming enzalutamide resistance by blocking each target individually or in combination needs to be tested.

### Intratumoral Androgen Biosynthesis

Intratumoral androgen biosynthesis has been recognized as one of the mechanisms of resistance to ADT and the development of CRPC (68–71). Elevated androgen levels were recently found in the bone marrow of patients treated with enzalutamide (72). An increase of intratumoral androgen biosynthesis is reported to be involved in enzalutamide resistance (73). Mechanistic studies revealed that several genes involved in the androgen synthesis pathway were significantly increased in enzalutamide-resistant PCa cells relative to the parental cells, including *AKR1C3* (aldo-keto reductase family 1 member C3) (73).

AKR1C3 is an enzyme that participates in the conversion of weak androgens such as androstenediones to more-active androgens such as testosterone or dihydrotestosterone. Knock-down of the *AKR1C3* gene or blocking of the AKR1C3 enzyme using a drug such as indomethacin can overcome enzalutamide resistance. Furthermore, overexpression of AKR1C3 can cause enzalutamide resistance in PCa cells (73). While this was a thorough study both *in vitro* and *in vivo*, validation from patient samples is needed to justify more effort in translating this work.

### Lineage Switching

When lung cancer is exposed to targeted therapies, such as epidermal growth factor receptor (EGFR) inhibitor, adenocarcinoma cells can switch from EGFR dependent to EGFR independent with expression of neuroendocrine lineage markers, or to cells that lack all *EGFR* expression (74, 75). Researchers of Dr. Swayers’ group applied this idea to determine whether lineage plasticity is a novel mechanism of enzalutamide resistance in PCa. They found that combined *TP53* and *RB1* alterations were significantly higher in disease progressed tumors from men under treatment with enzalutamide or abiraterone (an androgen synthesis inhibitor) (76). The combined knock-down of *TP53* and *RB1* in human PCa LNCaP/AR and CWR22Pc-EP cells did confer enzalutamide resistance *in vitro*. Further characterization of the cells revealed increased expression of neuroendocrine markers but decreased luminal cell markers in both of the double knock-down cells compared with their respective parental LNCaP/AR and CWR22Pc-EP luminal PCa cells, and the cells switch from AR dependent to AR independent. In PCa, lineage switching from adenocarcinoma to neuroendocrine PCa cells has been reported, but without complete understanding of the mechanism that drives the plasticity (77). A back-to-back publication showed that loss of *RB1* and *TP53* genes conferred the PCa lineage plasticity, metastasis, and enzalutamide resistance by developing a genetically engineered mouse model crossed with a *Pten* knockout mouse (78). Together, these data suggest lineage plasticity under selective pressure such as enzalutamide treatment. Finally, pluripotency transcription factor, SOX2, was identified to be required for the lineage plasticity and enzalutamide resistance by the loss of *TP53* and *RB1*. Based on these significant advances, the future directions are to investigate how to target SOX2, discover the mechanisms of enzalutamide-induced loss of *TP5*3 and *RB1*, and to determine whether these mechanisms are targetable.

## Contributions from the Tumor Microenvironment

Cancer is a systemic disease, and studies showed that paracrine factors such as hepatocyte growth factor, Wnts, basic fibroblast growth factor (or FGF2), and some cytokines from the microenvironment contribute to PCa initiation, progression, and metastasis (64, 79–83). Therefore, it is possible that paracrine factors influence the efficacy of enzalutamide treatment. The most studied and most easily targeted factor from the microenvironment is interleukin 6 (IL6) (84–88), which signals through the Janus kinase/signal transducer and activator of transcription 3 (JAK/STAT3) pathway. One of the functions of IL6/JAK/STAT3 in PCa cells is stimulating the expression of stemness/self-renewal genes. IL6/JAK/STAT3 also induces the expression of suppressor of cytokine signaling 3 (SOCS3), which in turn suppresses the transcriptional activity of IL6/JAK/STAT3.

Culig led a study (84) to determine the effect of long-term enzalutamide treatment in an inflammatory environment, studying PCa cells treated with enzalutamide plus IL6 for 3 weeks. They found this combination treatment suppressed self-renewal genes and AR target genes in PCa cells that had SOCS3 overexpression, but the AR target genes were induced in PCa cells with SOCS3 knocked out, suggesting that uncontrolled IL6/JAK/STAT3 transactivation of androgen signaling conferred enzalutamide resistance. This study showed that an inflammatory microenvironment could control the efficacy of enzalutamide by fine-tuning signaling of the inflammatory cytokine IL6 (84). Others reported that bicalutamide or enzalutamide could promote macrophage infiltration that subsequently enhances PCa invasion. The mechanistic studies showed that blocking AR signaling in macrophages increased the expression of CCL2 through inhibiting the expression of PIAS3 (protein inhibitor of STAT3) and subsequent increase of STAT3 phosphorylation (38). To overcome this effect, ASC-J9, the AR degrader, could directly inhibit the STAT3 phosphorylation and activation (38).

In PCa bone metastases, because the bone matrix is a reservoir rich in cytokines, we suspect that there are other cytokines that could also contribute to enzalutamide resistance, such as IL8, stromal cell-derived factor 1 (also known CXCL12), and granulocyte colony-stimulating factor, which have been identified in cancer drug resistance (89). On the other hand, the PCa bone metastatic lesions are results of dysregulated activities of osteoclasts and osteoblasts that were hijacked by cancer cells. However, we do not know whether and how osteoclasts or osteoblasts affect enzalutamide resistance. Targeting osteoclasts that resorb bones using zoledronic acid or denosumab is effective in palliation (5, 6). Metastasized PCa cells adhere in the osteoblast niche, developing into overt metastatic lesions (90–95). Will combining enzalutamide with approaches that target the proliferation or differentiation of osteoblasts or osteoclasts be better therapies? Will the effects of enzalutamide on osteoblasts or osteoclasts contribute to the drug resistance? We believe combination therapies using enzalutamide plus approaches that target the bone microenvironment (either paracrine factors or the bone cells themselves) might be able to prevent and/or overcome the enzalutamide resistant in mCRPC.

## Summary and Conclusion

Currently, enzalutamide is the best therapeutic drug for improving the survival of mCRPC patients. Due to their heterogeneity and plasticity, cancer cells have various mechanisms of drug resistance that we have begun to learn about, knowledge that will lead to targeted approaches to improving the efficacy of enzalutamide. The current mechanisms of enzalutamide resistance, the approaches for overcoming resistance, and the problems and solutions associated with these mechanisms are summarized in Table 1. The ultimate solution to drug resistance will be found only through future studies on the effects and mechanisms of enzalutamide action at the cellular and molecular levels.

**Table 1:** Summary of mechanisms and approaches of overcoming the enzalutamide resistance in preclinical PCa model.

Mechanisms of resistance	Targets	Approaches (drugs)	Mechanism of action	Problems and solutions
Constitutive AR signaling activation	AR-V	ASC-J9	Degrades ARs	Unknown other effects

		BET inhibitors (JQ1, OTX015)	Inhibits expression of ARs	Develop of resistance to BET inhibitor. Combination of BET inhibitor with CDK9 inhibition or/and PARP inhibitor

AR mutations	AR mutants	ASC-J9	Degrades ARs	Other effects unknown

	Transcriptional activities	Darolutamide (ODM-201)	AR antagonist	Unknown

	CDK4/6	CDK4/6 inhibitor (LEE011)	Inhibits AR-induced cell proliferation	Non-specificity (target all proliferative cells)

Bypassing AR signaling	GR	Arylpyrazole compound 15	GR antagonist	GR signaling is essential for normal functions, and GR antagonists activate AR

	To increase 11β-HSD2 expression or/and activity	Unknown	Converts cortisol (active form of GR ligands) to cortisone (inactive form of GR ligands)	Unknown

	AMFR (A ubiquitin E3-ligase)	Unknown	Degrades 11β-HSD2	Unknown

	Wnt5A	siRNA	Wnt5A knock-down	Unknown

	Autophagy	Clomipramine or metformin	Inhibits autophagy	Non-specificity

Intratumoral androgen synthesis	AKR1C3	Indomethacin	Inhibits conversion of weak androgens to more-active androgens	Validation in human

Lineage switching	SOX2	Unknown	Mediates the lineage plasticity by loss of *RB1* and *TP53*	Unknown

Microenvironmental effects	IL6/JAK/STAT3	ASC-J9	Inhibits STAT3 phosphorylation and activation	Other effects unknown

		IL6	Inhibits PCa cells with SOCS3 overexpression	Opposite effect in PCa cells without SOCS3 expression

*AR, androgen receptor; GR, glucocorticoid receptor; AMFR, autocrine mobility factor receptor; SOCS3, suppressor of cytokine signaling 3; JAK/STAT3, Janus kinase/signal transducer and activator of transcription 3; IL6, interleukin 6; 11β-HSD2, 11β-hydroxysteoid dehydrogenase-2; AKR1C3, aldo-keto reductase family 1 member C3; ASC-J9, 5-hydroxy-1,7-bis(3,4-dimethoxyphenyl)-1,4,6-heptatrien-3-one; LEE011, a small-molecule inhibitor for CDK4/6; PCa, prostate cancer; CDK, cyclin-dependent kinase*.

## Author Contributions

Designed, wrote, and finalized the manuscript: XL. Wrote parts of the manuscript: AA and JC.

## Conflict of Interest Statement

The authors declare that the research was conducted in the absence of any commercial or financial relationships that could be construed as a potential conflict of interest.
